# Supplementary material for: Zika virus pathogenesis in rhesus macaques is unaffected by pre-existing immunity to dengue virus
Source: Nat Commun. 2017 Jun 23;8:15674. doi: 10.1038/ncomms15674 (PMC5490051; doi:10.1038/ncomms15674)
Supplement: Supplementary Information — Supplementary Figures, Supplementary Tables, Supplementary Note, Supplementary Methods and Supplementary References. [file ncomms15674-s1.pdf]

**Supplementary Figures**

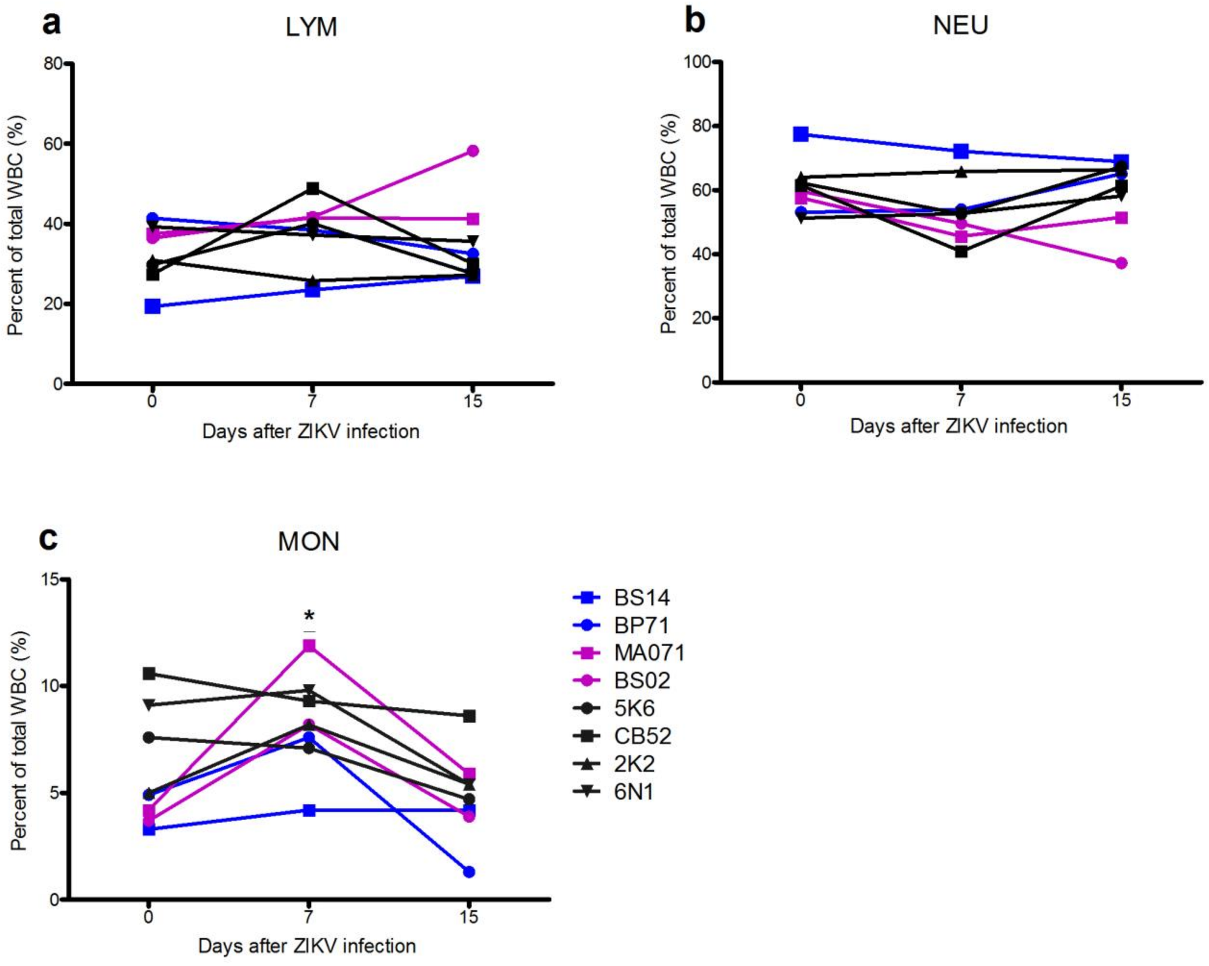


**Supplementary Figure 1. Kinetics of cell subsets:** (a-c) Cell subsets (LYM: Lymphocytes, NEU: Neutrophils, MON: Monocytes) kinetics obtained from Complete Blood Cell (CBC) counts (% of total WBC) performed at baseline and on days 7 and 15 p.i. are shown in percentage of total cells. Comparison of percentage of MON within cohorts on day 7 p.i. related to their own baseline values was performed using a two-tailed unpaired t test with Sidak-Bonferroni correction (*p<0.05*). In all panels, DENV-1-pre-exposed macaques are in blue, DENV-2 pre-exposed ones in magenta, and naïve ones in black.

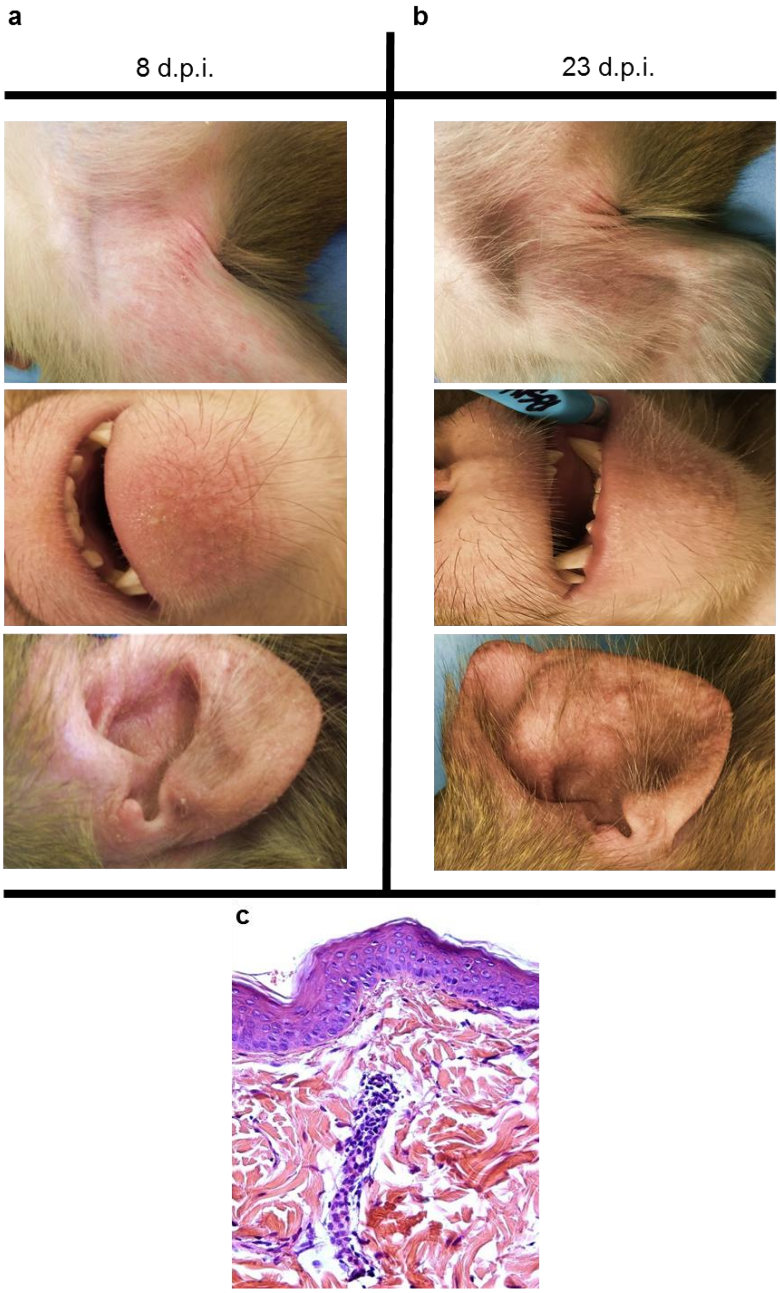


**Supplementary Figure 2: Non-pruritic skin rash developed by macaque BS14.** BS14 macaque from cohort 1 that was pre-exposed to DENV-1 shows, a) on day 8 p.i. of ZIKV, skin rash in several areas of the body such as axillaries, chin, and ears, and (b) by day 23 p.i., most of the rash was partially resolved in these areas. (c) Hematoxylin and eosin light micrograph of the haired skin of this macaque examined at 40x magnification. Few superficial dermal capillaries exhibit low numbers of perivascular mononuclear inflammatory infiltrate with a predominant population of lymphocytes but lesser plasma cells and histiocytes. Endothelial lining of capillaries is slightly hypertrophied and few dermal capillaries are surrounded by increased space with subtle separation of dermal collagen bundles adjacent to the vessels (edema).

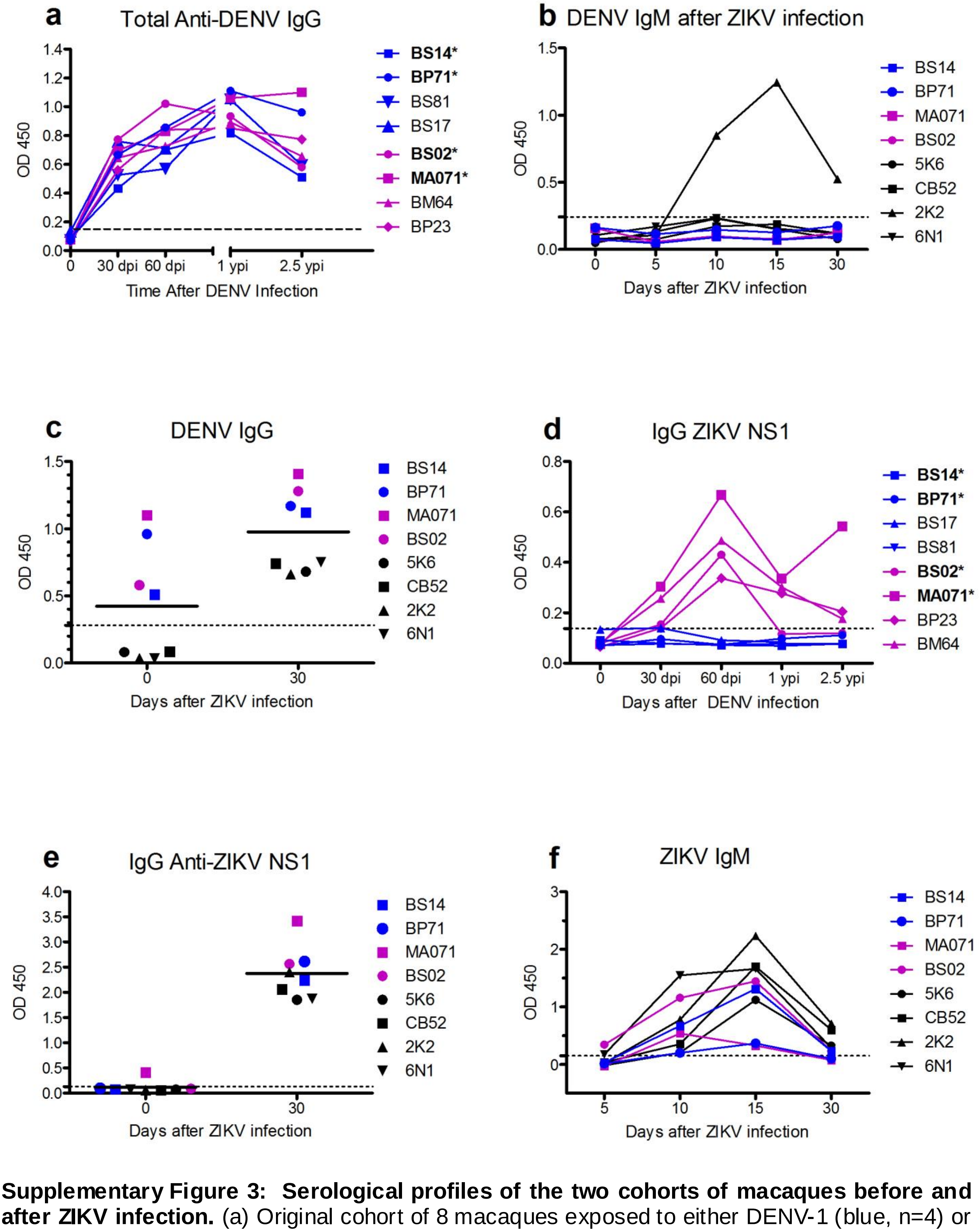


**Supplementary Figure 3: Serological profiles of the two cohorts of macaques before and after ZIKV infection.** (a) Original cohort of 8 macaques exposed to either DENV-1 (blue, n=4) or to DENV-2 (magenta, n=4) in the year 2013. All eight macaques showed an increase of DENV-IgG with high titers up to 2.5 y.p.i of DENV prior to ZIKV challenge. Two macaques per serotype

(marked with * in panel a) were selected to be challenged with ZIKV. (b) Both naïve and selected DENV-pre-exposed macaques were negative for DENV-IgM previous to ZIKV infection. As shown only one naïve macaque (2K2) developed cross-reacting IgM against DENV, as early as 10 d.p.i. of ZIKV. (c) DENV-naïve macaques were negative for DENV-IgG at baseline and both cohorts developed cross-reacting DENV-IgG after ZIKV infection. (d) Only macaques pre-exposed to DENV-2, but not those exposed to DENV-1, developed cross-reacting IgG to ZIKV-NS1 prior to ZIKV infection. (e) After ZIKV infection both cohorts have detectable IgG to ZIKV NS1 protein. (f) All macaques developed IgM to ZIKV at least by day 5 p.i. of ZIKV. By day 15 p.i., DENV-naïve macaques trend to have higher ZIKV-IgM levels compared to DENV-pre-exposed macaques. By day 30 p.i., IgM levels were back to basal levels.

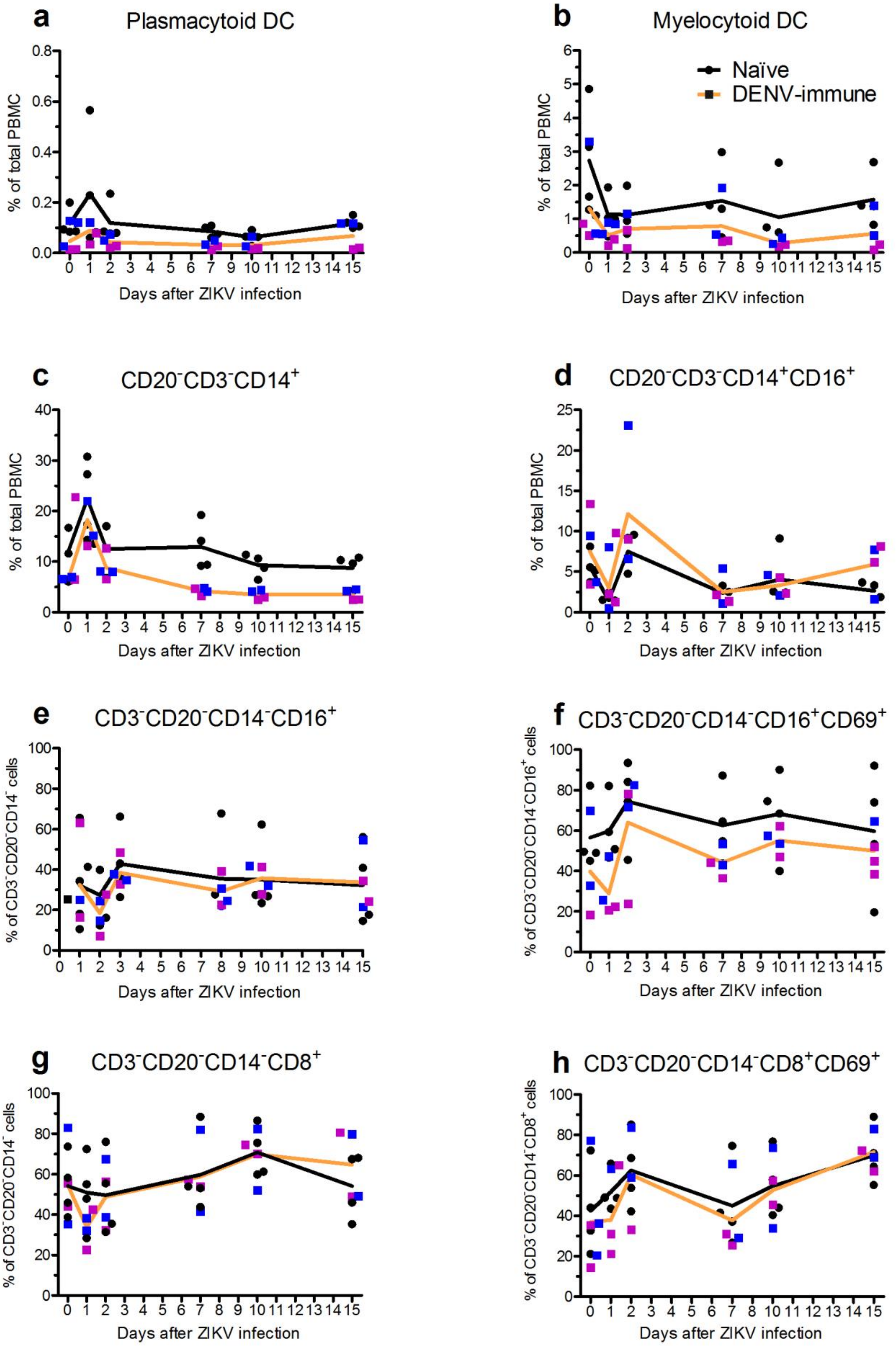

a
Plasmacytoid DC
% of total PBMC
Days after ZIKV infection
b
Myelocytoid DC
Naïve
DENV-immune
% of total PBMC
Days after ZIKV infection
c
$CD20^-CD3^-CD14^+$
% of total PBMC
Days after ZIKV infection
d
$CD20^-CD3^-CD14^+CD16^+$
% of total PBMC
Days after ZIKV infection
e
$CD3^-CD20^-CD14^-CD16^+$
% of $CD3^-CD20^-CD14^-$ cells
Days after ZIKV infection
f
$CD3^-CD20^-CD14^-CD16^+CD69^+$
% of $CD3^-CD20^-CD14^-CD16^+$ cells
Days after ZIKV infection
g
$CD3^-CD20^-CD14^-CD8^+$
% of $CD3^-CD20^-CD14^-$ cells
Days after ZIKV infection
h
$CD3^-CD20^-CD14^-CD8^+CD69^+$
% of $CD3^-CD20^-CD14^-CD8^+$ cells
Days after ZIKV infection

**Supplementary Figure 4: ZIKV does not induce significant changes in the plasmacytoid, myelocytoid dendritic cells, monocytes, and NK cell subsets.** (a) Plasmacytoid and (b) Myelocytoid cells were defined as HLA-DR$^{+}$CD3$^{-}$CD14$^{-}$CD20$^{-}$CD66$^{-}$CD123$^{+}$ and CD11c$^{+}$, respectively in PBMCs. No statistical differences were detected. (c) CD20$^{-}$CD3$^{-}$CD14$^{+}$ monocytes, (d) CD20$^{-}$CD3$^{-}$CD14$^{+}$CD16$^{+}$ monocytes, (e) NKCD16$^{+}$ cell subset and (f) its frequency of activation (CD69$^{+}$), and (g) NKCD8$^{+}$ cells subset and (h) its frequency of activation (CD69$^{+}$). Mean values are represented in orange and black lines for DENV-pre-exposed and naïve macaques, respectively. Black circles represent individual naïve macaques, and blue and magenta squares represent individuals previously exposed to either DENV-1 or DENV-2, respectively.

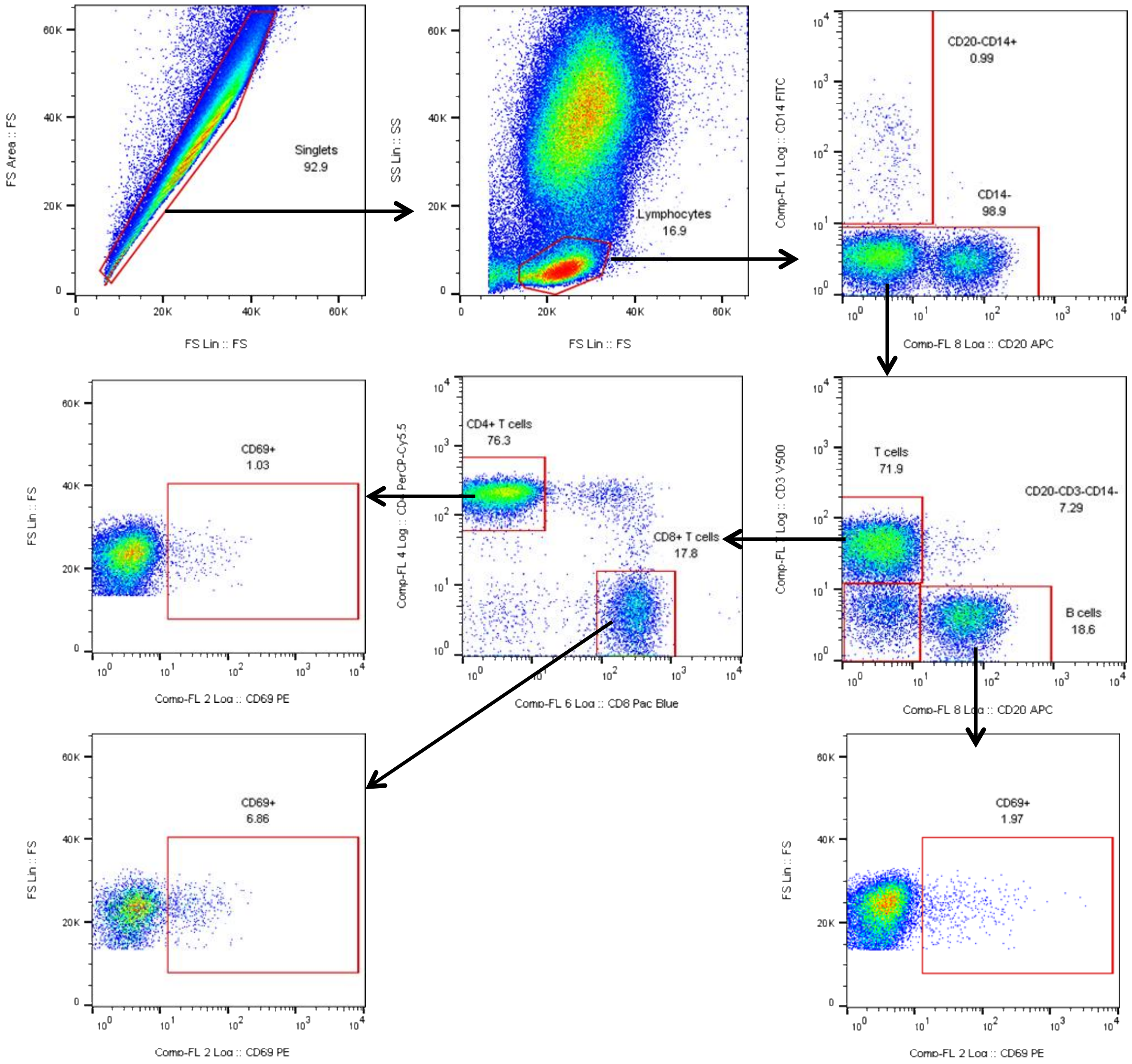


**Supplementary Figure 5: Gating strategy for B and T cells and defining subsets.** Characterization of T and B cells is shown. Gating for activation marker CD69 is also shown.

## Supplementary Tables

### Supplementary Table 1. ZIKV RNA detection in urine through 30 days post-infection

| RM ID | History | ZIKV RNA in Urine (Days after infection) | | | | | | | | | | | | Total Days/ Group |
|---|---|---|---|---|---|---|---|---|---|---|---|---|---|---|
| | | 1 | 3 | 5 | 7 | 9 | 15 | 17 | 19 | 21 | 23 | 25 | 30 | |
| | **Cohort 1** | | | | | | | | | | | | | |
| BS14 | $1^{o}$ DENV1 | **+++** | **-** | **-** | **+** | **+** | **-** | **-** | **+** | **-** | **-** | **-** | **-** | 10 |
| BP71 | $1^{o}$ DENV1 | **+++** | **-** | **+** | **-** | **-** | **-** | **-** | **-** | **-** | **-** | **-** | **-** | |
| MA0 71 | $1^{o}$ DENV2 | **-** | **-** | **-** | **+** | **+++** | **-** | **-** | **-** | **-** | **-** | **-** | **-** | |
| BS02 | $1^{o}$ DENV2 | **-** | **-** | **-** | **+** | **+** | **-** | **-** | **-** | **-** | **-** | **-** | **-** | |
| | **Cohort 2** | | | | | | | | | | | | | |
| 5K6 | Naïve | **-** | **+** | **-** | **-** | **-** | **-** | **-** | **-** | **-** | **-** | **-** | **-** | |
| CB52 | Naïve | **+** | **-** | **-** | **-** | **-** | **-** | **-** | **-** | **-** | **-** | **+** | **-** | |
| 2K2 | Naïve | **-** | **-** | **+** | **-** | **+++** | **-** | **-** | **+** | **-** | **-** | **-** | **-** | 9 |
| 6N1 | Naïve | **-** | **+** | **+** | **+++** | **-** | **-** | **-** | **-** | **-** | **-** | **-** | **-** | |

+++ Positive
+ Likely-Positive
- Negative

ZIKV RNA positive urine samples (+++, Ct: ≤40.64) using qRT-PCR were detected in both groups within the first 9 d.p.i. Same total of likely-positive samples (+) were detected in both groups (7 samples per group); these samples showed positive-like amplification curves over assay threshold, but their vRNA detection (Ct range: 40.75-41.65) was under the detection limit of the assay (Limited Ct: ≤40.64). Negative urine samples (-, no amplification).

**Supplementary Table 2 Percent positive stained of unstimulated controls**

| RM ID | History | Intracellular Staining: CD8[(1)] IFN-γ | CD8 TNF-α | CD8 CD107a | CD4[(2)] IFN-γ | CD4 TNF-α | CD4 CD107A |
|---|---|---|---|---|---|---|---|
| | **Cohort 1** | | | | | | |
| BS14 | 1° DENV-1 Day 30 | 0.20 | 0.48 | 1.27 | 0.11 | 0.11 | 0.46 |
| BP71 | 1° DENV-1 Day 30 | 0.68 | 0.62 | 1.87 | 0.24 | 0.46 | 0.71 |
| MA071 | 1° DENV-2 Day 30 | 0.38 | 0.49 | 0.34 | 0.18 | 0.16 | 0.21 |
| BS02 | 1° DENV-2 Day 30 | 0.28 | 0.47 | 0.62 | 0.10 | 0.19 | 0.28 |
| | **Cohort 2** | | | | | | |
| 5K6 | Naïve Day 30 | 0.19 | 0.73 | 0.89 | 0.10 | 0.23 | 1.01 |
| CB52 | Naïve Day 30 | 0.11 | 0.58 | 2.10 | 0.05 | 0.28 | 0.72 |
| 2K2 | Naïve Day 30 | 0.33 | 1.40 | 0.34 | 0.13 | 0.69 | 0.53 |
| 6N1 | Naïve Day 30 | 0.20 | 0.65 | 0.15 | 0.12 | 0.62 | 0.35 |

| RM ID | History | Intracellular Staining: CD8 IFN-γ | CD8 TNF-α | CD8 CD107a | CD4 IFN-γ | CD4 TNF-α | CD4 CD107A |
|---|---|---|---|---|---|---|---|
| | **Cohort 1** | | | | | | |
| BS14 | 1° DENV-1 Day 60 | 0.11 | 0.59 | 1.17 | 0.06 | 0.17 | 0.33 |
| BP71 | 1° DENV-1 Day 60 | 0.23 | 0.26 | 1.31 | 0.01 | 0.12 | 0.22 |
| MA071 | 1° DENV-2 Day 60 | 0.19 | 0.29 | 0.76 | 0.01 | 0.19 | 0.45 |
| BS02 | 1° DENV-2 Day 60 | 0.15 | 0.17 | 1.09 | 0.04 | 0.06 | 0.12 |
| | **Cohort 2** | | | | | | |
| 5K6 | Naïve Day 60 | 0.07 | 0.38 | 0.76 | 0.05 | 0.25 | 0.53 |
| CB52 | Naïve Day 60 | 0.02 | 0.11 | 0.46 | 0.02 | 0.04 | 0.18 |
| 2K2 | Naïve Day 60 | 0.06 | 0.12 | 0.51 | 0.02 | 0.22 | 0.13 |
| 6N1 | Naïve Day 60 | - | - | - | - | - | - |

[1]$CD8^+$ refers to $CD3^+CD20^-CD8^+$
[2]$CD4^+$ refers to $CD3^+CD20^-CD4^+$

Measurement of functional effector response of CD4+ and CD8+ T cells post ZIKV infection'. Antigen-specific CD4+ and CD8+ T cell effector responses were measured 30 d.p.i. of ZIKV to determine if prior DENV exposure impacted the ZIKV-specific functional response from these cells, and measured again after 60 d.p.i. to assess the stability of the functional effector response

## Supplementary Table 3 Dengue viremia post challenge in Cohort 1 animals

| RM ID | DENV Exposure History | Viremia[a] Days Post-Dengue Challenge ($log_{10}$ FFU/ml) 1 | 2 | 3 | 4 | 5 | 6 | 7 | 8 | 9 | 10 | CV[b] $log_{10}$ FFU/ml | Average Duration (Days) |
|---|---|---|---|---|---|---|---|---|---|---|---|---|---|
| BS14* | DENV-1 | - | 2.3 | 1.2 | - | - | - | - | - | - | - | 3.5 | 1.8 |
| BP71* | DENV-1 | - | 2.4 | 1.4 | - | - | - | - | - | - | - | 3.8 | |
| BS17 | DENV-1 | - | - | 0.9 | | | | | | | | 0.9 | |
| BS81 | DENV-1 | - | 1.8 | 1.4 | - | - | - | - | - | - | - | 3.2 | |
| MA071* | DENV-2 | 1.2 | 1.8 | 1.5 | 1.5 | - | - | - | - | - | - | 6.1 | 5 |
| BS02* | DENV-2 | 0.9 | 1.5 | - | 0.9 | 1.2 | 2 | 1.8 | 1.2 | - | - | 9.6 | |
| BP23 | DENV-2 | - | 1.9 | 2.2 | 2.5 | 1.4 | - | - | - | - | - | 7.9 | |
| BM64 | DENV-2 | - | 2.1 | 1.4 | 1.5 | 2.2 | 1.6 | - | - | - | - | 8.8 | |

*Animals included in Cohort 1 of this work.

a) Viremia was quantified by immunofocus assay, which measured infectious virus in Vero cells. (-) Indicated below the level of detection of 8 PFU/ml.

b) CV (Cumulative viremia) is defined as the total daily viremia for each animal

**Supplementary Table 4**

Dengue Viremia post Zika infection

| RM ID | DENV Exposure History | Viremia (Qualitative) Days Post-ZIKV Infection | | | | | | | | | |
|---|---|---|---|---|---|---|---|---|---|---|---|
| | | 1 | 2 | 3 | 4 | 5 | 6 | 7 | 8 | 9 | 10 |
| | **Cohort 1** | | | | | | | | | | |
| BS14 | 1° DENV-1 | - | - | - | - | - | - | - | - | - | - |
| BP71 | 1° DENV-1 | - | - | - | - | - | - | - | - | - | - |
| MA071 | 1° DENV-2 | - | - | - | - | - | - | - | - | - | - |
| BS02 | 1° DENV-2 | - | - | - | - | - | - | - | - | - | - |

For DENV1, DENV-1WP74 RNA was used as a positive control which amplified at a treshold cycle (Ct)= 11.77.
For DENV2, DENV-2 NGC RNA was used as a positive control which amplified at Ct=13.94.
Samples are considered positive when amplification occurs at Ct<36.

**Supplementary Table 5 Primers and Probe for Zika RT-PCR**

| Primer/Probe | Sequence |
|---|---|
| ZIKV 1086f | 5’ CCG CTG CCC AAC ACA AG 3’ |
| ZIKV 1162c | 5’ CCA CTA ACG TTC TTT TGC AGA CAT 3’ |
| ZIKV 1107 | FAM 5’ AGC CTA CCT TGA CAA GCA GTC AGA CAC TCA A 3’ (BHQ-1) |

**Supplementary Table 6 Antibodies for Cell Phenotyping**

| Phenotype/activation | Company | Catalog number | Dilution Total ul in 160 ul final volume |
|---|---|---|---|
| CD14 FITC | Beckman-Coulter | 6603262 | 4 |
| CD69 PE | DAKO | R7173 | 5 |
| CD4 PerCP-Cy5.5 | BD-Biosciences | 552838 | 5 |
| CD8 Pac Blue | Fisher-Invitrogen | MHCD0828 | 2.5 |
| CD3 V500 | BD-Biosciences | 560770 | 3 |
| CD20 APC | Biolegend | 302310 | 20 |
| CD16 Alexa700 | Biolegend | 560713 | 4 |
| **DC control** | | | |
| CD14 FITC | Beckman-Coulter | 6603262 | 4 |
| CD66abcd | Miltenyi | 130-093-132 | 5 |
| CD20 FITC | Beckman-Coulter | 6602381 | 1 |
| CD8 FITC | Fisher-Invitrogen | MHCD0801 | 2.5 |
| CD3 FITC | BD-Biosciences | 556611 | 7 |
| Isotype PE | Fisher-Invitrogen | MG104 | 1.5 |
| HLA-DR PerCP-Cy5.5 | BD-Biosciences | 552764 | 5 |
| Isotype APC | BD-Biosciences | 554681 | 1.5 |
| **DC tube** | | | |
| CD14 FITC | Beckman-Coulter | 6603262 | 4 |
| CD66abcd | Miltenyi | 130-093-132 | 5 |
| CD20 FITC | Beckman-Coulter | 6602381 | 1 |
| CD8 FITC | Fisher-Invitrogen | MHCD0801 | 2.5 |
| CD3 FITC | BD-Biosciences | 556611 | 7 |
| CD11c PE | Biolegend | 301606 | 7.5 |
| HLA-DR PerCP-Cy5.5 | BD-Biosciences | 552764 | 5 |
| CD123 APC | BD-Biosciences | 560087 | 10 |
| **NK markers** | | | |
| CD14 FITC | Beckman-Coulter | 6603262 | 4 |
| CD20 FITC | Beckman-Coulter | 6602381 | 1 |
| CD159a (NKG2A) PE | Beckman-Coulter | IM3291U | 8 |
| CD337 (NKp30) PC5 | Beckman-Coulter | PN A66904 | 5 |
| CD335 (NKp46) PC7 | Beckman-Coulter | PN B38703 | 3 |
| CD159c (NKG2C) APC* | R&D Systems | MAB1381-100 | 5 |
| CD16 Alx700 | Biolegend | 560713 | 4 |
| CD8 Pacific Blue | Fisher-Invitrogen | MHCD0828 | 2.5 |
| CD3 V500 | BD-Biosciences | 560770 | 5 |

*In house labeled

**Supplementary Table 7 Characterization of fluorochromes antibody panels and cytometer configurations for Natural Killer cells**

| | Pac Blue | V500 | FITC | PE | PerCP-Cy5.5 | PE-Cy7 | APC | AF700/APC-Cy7 |
|---|---|---|---|---|---|---|---|---|
| **Phenotyping** | CD8 | CD3 | CD14 | CD69 | CD4 | - | CD20 | CD16 |
| **NK markers** | CD8 | CD3 | CD14, CD20 | NKG2A | NKp30 | NKp46 | NKG2C | CD16 |
| **DC control** | - | - | CD3, CD8, CD14,CD20, CD66abcd | IgG | HLA-DR | - | IgG | - |
| **DC** | - | - | CD3, CD8, CD14,CD20, CD66abcd | CD11c | HLA-DR | - | CD123 | - |

Cells were defined as $CD3^{-}CD20_{-}CD14^{-}$ and analyzed for the expression of NK cell markers CD16, CD8, NKG2A, NKG2C, NKp30 and NKp46.

**Supplementary Table 8 Antibodies used for cell-immune response assessment**

| Marker | Stain | Clone | Catalog Number | Vendor | Dilution |
|---|---|---|---|---|---|
| CD4 | PerCP-Cy-5.5 | SK3 | 566316 | BD Biosciences | 1:25 |
| CD8β | PE | ECD | 6607123 | Beckman-Coulter | 1:20 |
| CD3 | Pacific Blue | SP34-2 | 558124 | BD Biosciences | 1:30 |
| CD20 | BV605 | 2H7 | 563783 | BD Biosciences | 1:30 |
| CD107a | FITC | H4A3 | 555800 | BD Biosciences | 1:10 |
| CD28 | PE-Cy-5 | CD28.2 | 555730 | BD Biosciences | 1:10 |
| CD95 | BV510 | DX2 | 305640 | Biolegend | 1:30 |
| IFN-γ | APC | B27 | 554702 | BD Biosciences | 1:30 |
| TNF-α | PE-Cy-7 | Mab11 | 557647 | BD Biosciences | 1:30 |

**Supplementary Table 9 Peptides sequences used to stimulate PBMCs 30 and 60 days after Zika infection**

## Dengue Virus Type 1 Peptides

| Peptide | Amino Acid Sequence | Peptide | Amino Acid Sequence | Peptide | Amino Acid Sequence |
|---|---|---|---|---|---|
| 1 | MRCVGIGNRDFVEGLSG | 29 | TTATITPQAPTSEIQLT | 57 | KYEGTDAPCKIPFSSQD |
| 2 | GNRDFVEGLSGATWVDV | 30 | PQAPTSEIQLTDYGALT | 58 | APCKIPFSSQDEKGVTQ |
| 3 | VEGLSGATWVDVVLEHG | 31 | SEIQLTDYGALTLDCSPR | 59 | FSSQDEKGVTQNGRLITA |
| 4 | ATWVDVVLEHGSCVTTM | 32 | YGALTLDCSPRTGLDFN | 60 | KGVTQNGRLITANPIVTD |
| 5 | VLEHGSCVTTMAKDKPT | 33 | DCSPRTGLDFNEMVLLT | 61 | RLITANPIVTDKEKPVN |
| 6 | CVTTMAKDKPTLDIELL | 34 | GLDFNEMVLLTMEKKSW | 62 | PIVTDKEKPVNIEAE |
| 7 | KDKPTLDIELLKTEVTN | 35 | MVLLTMEKKSWLVHKQW | 63 | DKEKPVNIEAEPPFGE |
| 8 | DIELLKTEVTNPAVLRK | 36 | EKKSWLVHKQWFLDLPL | 64 | VNIEAEPPFGESYIVVG |
| 9 | TEVTNPAVLRKLCIEAK | 37 | VHKQWFLDLPLPWTSGA | 65 | PPFGESYIVVGAGEKAL |
| 10 | AVLRKLCIEAKISNTTT | 38 | LDLPLPWTSGASTSQET | 66 | YIVVGAGEKALKLSWFK |
| 11 | CIEAKISNTTTDSRCPT | 39 | WTSGASTSQETWNRQDL | 67 | GEKALKLSWFKKGSSIG |
| 12 | SNTTTDSRCPTQGEATL | 40 | TSQETWNRQDLLVTFKT | 68 | KLSWFKKGSSIGKMFEA |
| 13 | SRCPTQGEATLVEEQDT | 41 | NRQDLLVTFKTAHAKKQ | 69 | KGSSIGKMFEATARGAR |
| 14 | GEATLVEEQDTNFVCRR | 42 | VTFKTAHAKKQEVVVLG | 70 | KMFEATARGARRMAILG |
| 15 | VEEQDTNFVCRRTFVDR | 43 | HAKKQEVVVLGSQEGAM | 71 | ARGARRMAILGDTAWDF |
| 16 | NFVCRRTFVDRGWGNG | 44 | VVVLGSQEGAMHTALTG | 72 | MAILGDTAWDFGSIGGV |
| 17 | RTFVDRGWGNGCGLFGK | 45 | SQEGAMHTALTGATEIQ | 73 | TAWDFGSIGGVFTSVGK |
| 18 | GWGNGCGLFGKGSLIT | 46 | HTALTGATEIQTSGTTT | 74 | SIGGVFTSVGKLIHQIF |
| 19 | CGLFGKGSLITCAKFK | 47 | ATEIQTSGTTTIFAGHL | 75 | TSVGKLIHQIFGTAYGV |
| 20 | KGSLITCAKFKCVTKLE | 48 | SGTTTIFAGHLKCRLKM | 76 | IHQIFGTAYGVLFSGV |
| 21 | CAKFKCVTKLEGKIVQY | 49 | FAGHLKCRLKMDKLTLK | 77 | GTAYGVLFSGVSWTMKI |
| 22 | VTKLEGKIVQYENLKY | 50 | CRLKMDKLTLKGMSYVM | 78 | LFSGVSWTMKIGIGILL |
| 23 | GKIVQYENLKYSVIVTV | 51 | KLTLKGMSYVMCTGSFK | 79 | WTMKIGIGILLTWLGLN |
| 24 | YENLKYSVIVTVHTGDQ | 52 | MSYVMCTGSFKLEKEVA | 80 | IGILLTWLGLNSRSTSL |
| 25 | SVIVTVHTGDQHQVGNE | 53 | TGSFKLEKEVAETQHGT | 81 | WLGLNSRSTSLSMTCIA |
| 26 | HTGDQHQVGNETTEHGT | 54 | LEKEVAETQHGTVLVQV | 82 | RSTSLSMTCIAVGMVTL |
| 27 | HQVGNETTEHGTTATIT | 55 | AETQHGTVLVQVKYEGT | 83 | MTCIAVGMVTLYLGVMV |
| 28 | TTEHGTTATITPQAPT | 56 | TVLVQVKYEGTDAPCKI | 84 | GMVTLYLGVMVQA |

## Supplementary Table 9 Cont

### Dengue Virus Type 2 Peptides

| Peptide | Amino Acid Sequence | Peptide | Amino Acid Sequence | Peptide | Amino Acid Sequence |
|---|---|---|---|---|---|
| 1 | MRCIGISNRDFVEGV | 29 | AWLVHRQWFLDLPLPWL | 57 | MRGAKRMAILGDTAWDF |
| 2 | ISNRDFVEGVSGGSWVDI | 30 | WFLDLPLPWLPGADTQGSNW | 58 | AILGDTAWDFGSLGGVF |
| 3 | GVSGGSWVDIVLEHGSCV | 31 | PGADTQGSNWIQKETLV | 59 | WDFGSLGGVFTSIGKAL H |
| 4 | DIVLEHGSCVTTMAKNK | 32 | SNWIQKETLVTFKNPHAK | 60 | VFTSIGKALHQVFGAIY |
| 5 | SCVTTMAKNKPTLDFELI | 33 | LVTFKNPHAKKQDVVVL | 61 | ALHQVFGAIYGAAFSGV |
| 6 | NKPTLDFELIETEAKQPA | 34 | HAKKQDVVVLGSQEGAMH | 62 | AIYGAAFSGVSWIMKILI |
| 7 | LIETEAKQPATLRKYCI | 35 | VLGSQEGAMHTALTGA | 63 | GVSWIMKILIGVIITWI |
| 8 | KQPATLRKYCIEAKL | 36 | GAMHTALTGATEIQM | 64 | ILIGVIITWIGMNSR |
| 9 | LRKYCIEAKLTNTTTDSR | 37 | ALTGATEIQMSSGNLLF | 65 | IITWIGMNSRSTSLSVSL |
| 10 | KLTNTTTDSRCPTQGEPSL | 38 | IQMSSGNLLFTGHLKCRL | 66 | SRSTSLSVSLVLVGVVTL |
| 11 | RCPTQGEPSLNEEQDKRF | 39 | LFTGHLKCRLRMDKLQLK | 67 | SLVLVGVVTLYLGVMVQ A |
| 12 | SLNEEQDKRFVCKHSMV | 40 | RLRMDKLQLKGMSYSM | | |
| 13 | KRFVCKHSMVDRGWGNG CGL | 41 | LQLKGMSYSMCTGKFKVV | | |
| 14 | DRGWGNGCGLFGKGGIV | 42 | SMCTGKFKVVKEIAETQH | | |
| 15 | CGLFGKGGIVTCAMFTCK | 43 | VVKEIAETQHGTIVIRV | | |
| 16 | IVTCAMFTCKKNMKGKVV | 44 | TQHGTIVIRVQYEGDGSPCK | | |
| 17 | CKKNMKGKVVQPENLEY | 45 | VQYEGDGSPCKIPFEIM | | |
| 18 | KVVQPENLEYTIVITPH | 46 | SPCKIPFEIMDLEKRHVL | | |
| 19 | LEYTIVITPHSGEEHAV | 47 | IMDLEKRHVLGRLITV | | |
| 20 | TPHSGEEHAVGNDTGKH | 48 | RHVLGRLITVNPIVTEK | | |
| 21 | HAVGNDTGKHGKEIKI | 49 | ITVNPIVTEKDSPVNIEA | | |
| 22 | TGKHGKEIKITPQSSI | 50 | EKDSPVNIEAEPPFGDSY | | |
| 23 | EIKITPQSSITEAELTGY | 51 | EAEPPFGDSYIIIGV | | |
| 24 | SITEAELTGYGTVTM | 52 | FGDSYIIIGVEPGQLKL | | |
| 25 | ELTGYGTVTMECSPRTGL | 53 | IGVEPGQLKLNWFKK | | |
| 26 | TMECSPRTGLDFNEMVLL | 54 | GQLKLNWFKKGSSIGQMI | | |
| 27 | GLDFNEMVLLQMENKAWL | 55 | KKGSSIGQMIETTMRGAK | | |
| 28 | LLQMENKAWLVHRQWFL | 56 | MIETTMRGAKRMAIL | | |

## Supplementary Table 9 Cont

### Zika Virus Envelope Peptides

| Peptide | Amino Acid Sequence | Peptide | Amino Acid Sequence | Peptide | Amino Acid Sequence |
|---|---|---|---|---|---|
| ZIKV59 | IRCIGVSNRDFVEGM | ZIKV87 | LSVHGSQHSGMIVND | ZIKV115 | KGRLSSGHLKCRLKM |
| ZIKV60 | VSNRDFVEGMSGGTW | ZIKV88 | SQHSGMIVNDTGHET | ZIKV116 | SGHLKCRLKMDKLRL |
| ZIKV61 | FVEGMSGGTWVDVVL | ZIKV89 | MIVNDTGHETDENRA | ZIKV117 | CRLKMDKLRLKGVSY |
| ZIKV62 | SGGTWVDVVLEHGGC | ZIKV90 | TGHETDENRAKVEIT | ZIKV118 | DKLRLKGVSYSLCTA |
| ZIKV63 | VDVVLEHGGCVTVMA | ZIKV91 | DENRAKVEITPNSPR | ZIKV119 | KGVSYSLCTAAFTFT |
| ZIKV64 | EHGGCVTVMAQDKPT | ZIKV92 | KVEITPNSPRAEATL | ZIKV120 | SLCTAAFTFTKIPAE |
| ZIKV65 | VTVMAQDKPTVDIEL | ZIKV93 | PNSPRAEATLGGFGS | ZIKV121 | AFTFTKIPAETLHGT |
| ZIKV66 | QDKPTVDIELVTTTV | ZIKV94 | AEATLGGFGSLGLDC | ZIKV122 | KIPAETLHGTVTVEV |
| ZIKV67 | VDIELVTTTVSNMAE | ZIKV95 | GGFGSLGLDCEPRTG | ZIKV123 | TLHGTVTVEVQYAGT |
| ZIKV68 | VTTTVSNMAEVRSYC | ZIKV96 | LGLDCEPRTGLDFSD | ZIKV124 | VTVEVQYAGTDGPCK |
| ZIKV69 | SNMAEVRSYCYEASI | ZIKV97 | EPRTGLDFSDLYYLT | ZIKV125 | QYAGTDGPCKVPAQM |
| ZIKV70 | VRSYCYEASISDMAS | ZIKV98 | LDFSDLYYLTMNNKH | ZIKV126 | DGPCKVPAQMAVDMQ |
| ZIKV71 | YEASISDMASDSRCP | ZIKV99 | LYYLTMNNKHWLVHK | ZIKV127 | VPAQMAVDMQTLTPV |
| ZIKV72 | SDMASDSRCPTQGEA | ZIKV100 | MNNKHWLVHKEWFHD | ZIKV128 | AVDMQTLTPVGRLIT |
| ZIKV73 | DSRCPTQGEAYLDKQ | ZIKV101 | WLVHKEWFHDIPLPW | ZIKV129 | TLTPVGRLITANPVI |
| ZIKV74 | TQGEAYLDKQSDTQY | ZIKV102 | EWFHDIPLPWHAGAD | ZIKV130 | GRLITANPVITESTE |
| ZIKV75 | YLDKQSDTQYVCKRT | ZIKV103 | IPLPWHAGADTGTPH | ZIKV131 | ANPVITESTENSKMM |
| ZIKV76 | SDTQYVCKRTLVDRG | ZIKV104 | HAGADTGTPHWNNKE | ZIKV132 | TESTENSKMMLELDP |
| ZIKV77 | VCKRTLVDRGWGNGC | ZIKV105 | TGTPHWNNKEALVEF | ZIKV133 | NSKMMLELDPPFGDS |
| ZIKV78 | LVDRGWGNGCGLFGK | ZIKV106 | WNNKEALVEFKDAHA | ZIKV134 | LELDPPFGDSYIVIG |
| ZIKV79 | WGNGCGLFGKGSLVT | ZIKV107 | ALVEFKDAHAKRQTV | ZIKV135 | PFGDSYIVIGVGEKK |
| ZIKV80 | GLFGKGSLVTCAKFA | ZIKV108 | KDAHAKRQTVVVLGS | ZIKV136 | YIVIGVGEKKITHHW |
| ZIKV81 | GSLVTCAKFACSKKM | ZIKV109 | KRQTVVVLGSQEGAV | ZIKV137 | VGEKKITHHWHRSGS |
| ZIKV82 | CAKFACSKKMTGKSI | ZIKV110 | VVLGSQEGAVHTALA | ZIKV138 | ITHHWHRSGSTIGKA |
| ZIKV83 | CSKKMTGKSIQPENL | ZIKV111 | QEGAVHTALAGALEA | ZIKV139 | HRSGSTIGKAFEATV |
| ZIKV84 | TGKSIQPENLEYRIM | ZIKV112 | HTALAGALEAEMDGA | ZIKV140 | TIGKAFEATVRGAKR |
| ZIKV85 | QPENLEYRIMLSVHG | ZIKV113 | GALEAEMDGAKGRLS | ZIKV141 | FEATVRGAKRMAVLG |
| ZIKV86 | EYRIMLSVHGSQHSG | ZIKV114 | EMDGAKGRLSSGHLK | ZIKV142 | RGAKRMAVLGDTAWD |

**Supplementary Table 9 Cont**

| Peptide | Amino Acid Sequence |
|---|---|
| ZIKV143 | MAVLGDTAWDFGSVG |
| ZIKV144 | DTAWDFGSVGGALNS |
| ZIKV145 | FGSVGGALNSLGKGI |
| ZIKV146 | GALNSLGKGIHQIFG |
| ZIKV147 | LGKGIHQIFGAAFKS |
| ZIKV148 | HQIFGAAFKSLFGGM |
| ZIKV149 | AAFKSLFGGMSWFSQ |
| ZIKV150 | LFGGMSWFSQILIGT |
| ZIKV151 | SWFSQILIGTLLMWL |
| ZIKV152 | ILIGTLLMWLGLNTK |
| ZIKV153 | LLMWLGLNTKNGSIS |
| ZIKV154 | GLNTKNGSISLMCLA |
| ZIKV155 | NGSISLMCLALGGVL |
| ZIKV156 | LMCLALGGVLIFLST |
| ZIKV157 | LGGVLIFLSTAVSAD |
| ZIKV158 | IFLSTAVSADVGCSV |
| ZIKV159 | AVSADVGCSVDFSKK |

## Supplementary Table 9 Cont

### Zika Virus NS1 Peptides

| Peptide | Amino Acid Sequence | Peptide | Amino Acid Sequence | Peptide | Amino Acid Sequence |
|---|---|---|---|---|---|
| ZIKV160 | VGCSVDFSKKETRCG | ZIKV188 | ECPLKHRAWNSFLVE | ZIKV216 | GTKVHVEETCGTRGP |
| ZIKV161 | DFSKKETRCGTGVFV | ZIKV189 | HRAWNSFLVEDHGFG | ZIKV217 | VEETCGTRGPSLRST |
| ZIKV162 | ETRCGTGVFVYNDVE | ZIKV190 | SFLVEDHGFGVFHTS | ZIKV218 | GTRGPSLRSTTASGR |
| ZIKV163 | TGVFVYNDVEAWRDR | ZIKV191 | DHGFGVFHTSVWLKV | ZIKV219 | SLRSTTASGRVIEEW |
| ZIKV164 | YNDVEAWRDRYKYHP | ZIKV192 | VFHTSVWLKVREDYS | ZIKV220 | TASGRVIEEWCCREC |
| ZIKV165 | AWRDRYKYHPDSPRR | ZIKV193 | VWLKVREDYSLECDP | ZIKV221 | VIEEWCCRECTMPPL |
| ZIKV166 | YKYHPDSPRRLAAAV | ZIKV194 | REDYSLECDPAVIGT | ZIKV222 | CCRECTMPPLSFRAK |
| ZIKV167 | DSPRRLAAAVKQAWE | ZIKV195 | LECDPAVIGTAVKGK | ZIKV223 | TMPPLSFRAKDGCWY |
| ZIKV168 | LAAAVKQAWEDGICG | ZIKV196 | AVIGTAVKGKEAVHS | ZIKV224 | SFRAKDGCWYGMEIR |
| ZIKV169 | KQAWEDGICGISSVS | ZIKV197 | AVKGKEAVHSDLGYW | ZIKV225 | DGCWYGMEIRPRKEP |
| ZIKV170 | DGICGISSVSRMENI | ZIKV198 | EAVHSDLGYWIESEK | ZIKV226 | GMEIRPRKEPESNLV |
| ZIKV171 | ISSVSRMENIMWRSV | ZIKV199 | DLGYWIESEKNDTWR | ZIKV227 | PRKEPESNLVRSMVT |
| ZIKV172 | RMENIMWRSVEGELN | ZIKV200 | IESEKNDTWRLKRAH | ZIKV228 | ESNLVRSMVTAGSTD |
| ZIKV173 | MWRSVEGELNAILEE | ZIKV201 | NDTWRLKRAHLIEMK | ZIKV229 | RSMVTAGSTDHMDHF |
| ZIKV174 | EGELNAILEENGVQL | ZIKV202 | LKRAHLIEMKTCEWP | | |
| ZIKV175 | AILEENGVQLTVVVG | ZIKV203 | LIEMKTCEWPKSHTL | | |
| ZIKV176 | NGVQLTVVVGSVKNP | ZIKV204 | TCEWPKSHTLWTDGI | | |
| ZIKV177 | TVVVGSVKNPMWRGP | ZIKV205 | KSHTLWTDGIEESDL | | |
| ZIKV178 | SVKNPMWRGPQRLPV | ZIKV206 | WTDGIEESDLIIPKS | | |
| ZIKV179 | MWRGPQRLPVPVNEL | ZIKV207 | EESDLIIPKSLAGPL | | |
| ZIKV180 | QRLPVPVNELPHGWK | ZIKV208 | IIPKSLAGPLSHHNT | | |
| ZIKV181 | PVNELPHGWKAWGKS | ZIKV209 | LAGPLSHHNTREGYR | | |
| ZIKV182 | PHGWKAWGKSYFVRA | ZIKV210 | SHHNTREGYRTQMKG | | |
| ZIKV183 | AWGKSYFVRAAKTNN | ZIKV211 | REGYRTQMKGPWHSE | | |
| ZIKV184 | YFVRAAKTNNSFVVD | ZIKV212 | TQMKGPWHSEELEIR | | |
| ZIKV185 | AKTNNSFVVDGDTLK | ZIKV213 | PWHSEELEIRFEECP | | |
| ZIKV186 | SFVVDGDTLKECPLK | ZIKV214 | ELEIRFEECPGTKVH | | |
| ZIKV187 | GDTLKECPLKHRAWN | ZIKV215 | FEECPGTKVHVEETC | | |

**Supplementary Note 1**

DENV-immune macaques (BS14) developed a nonpruritic skin rash that were centered on lightly haired skin areas (chin, axillary, ears, and inguinal areas) on day 8 p.i. (Supplementary Fig. 2a). This animal did not have a known history of allergy or skin condition. By day 23 p.i. most areas had self-resolved themselves except in the axillary and inguinal areas (Supplementary Fig. 2b). We performed diagnostic testing on affected areas by skin scraping for parasites, skin swabs for bacterial culture, samples for fungal cultures, and impression smears for cytology. We also examined skin biopsies from axillary and inguinal areas by qRT-PCR. Fungal cultures were negative and bacterial cultures showed only normal skin flora. Cytology indicated there was a neutrophilic and plasmacytic dermatitis with hyperkeratosis (Supplementary Fig. 2c). Histopathology of haired skin had a few number of lymphocytes, plasma cells, and lesser macrophages within the superficial dermis with predominant perivascular distribution. There was mild to moderate diffuse orthokeratotic hyperkeratosis (Supplementary Fig. 2d). No other abnormal clinical sign was noted in this animal during this period. qRT-PCR for ZIKV RNA from skin biopsy samples also did not show any specific amplification. To rule out the unlikely event of latent dengue reactivation as cause of this rash we tested the serum samples from the first ten days after ZIKV infection for the presence of dengue viremia in all four cohort 1 animals. Dengue RNA was not detected at any time after ZIKV challenge (Supplementary Table 4 and Supplementary Method)

## Supplementary Methods

### Dengue RT-PCR

Viral RNA from serum samples was extracted using QIAmp Viral RNA mini kit (Qiagen, Valencia, CA) according to manufacturer instructions. Real-time RT-PCR (TaqMan) singleplex assay- Specific primers and probes for DENV1: Forward (5’ CAA AAG GAA GTC GTG CAA TA 3’), Reverse (5’ CTG AGT GAA TTC TCT CTA CTG AAC C 3’), Probe (5’ CAT GTG GTT GGG AGC ACG C 3’ FAM/BHQ1) and DENV2: Forward (5’ CAG GTT ATG GCA CTG TCA CGA T 3’), Reverse (5’ CCA TCT GCA GCA ACA CCA TCT C 3’) and Probe (5’ CTC TCC GAG AAC AGG CCT CGA CTT CAA 3’ HEX/BHQ1) (Sigma-Aldrich) were used. RNA from known DENV1 or DENV2 was included as positive control. For the reaction mixture, 10uL of RNA was combined with 100uM primers and probes in a 50uL total volume using qScript™ One-Step qRTPCR master mix kit (Quanta Biosciences™) according to manufacturers instructions. Thermocycling parameters were as follows: Reverse transcription for 30min at 50°C and reverse transcriptase inactivation for 12:30min. at 95°C was followed by 45 cycles of 95°C for 15 seconds and annealing at 60°C for 1 min using the iCycler IQ5 Real Time Detection System (Optical System software version 2.1; Bio Rad, CA) [1].
Interpretation: Cycle threshold (Ct) is placed above background signal, usually intersecting the initial exponential phase of the amplification curve for each sample. Curves with treshold >36 render erratically and are difficult to determine as Ct values increases. Results may be unreliable, hence considered negative [1,2].

### Dengue viremia by immunofocus assay

Animals in cohort 1 were previously challenge with DENV1 or DENV2 in October 2013. After the challenge infectious virus in the blood was determined in serum samples within 10 days of challenge. As previously described [3] monolayer of Vero-81 cells were seeded in 24-well plates and inoculated with 100ul of challenge diluted serum samples (1:2.5, 1:5, 1:10, 1:20, 1:40) in triplicate wells. Cells were incubated for 1hr at 37C, then overlaid with 1ml of 1% methylocellulose Optic-MEM (Gibco) supplemented with 2% FBS (Cellgro) and antibiotics. After 4-5 days in a 37C incubator, cells were washed and fixed in 80% methanol. Immunocyto289 staining of DENV infected cells were assessed using anti-flavivirus monoclonal antibody 4G2 followed by a secondary antibody, HRP-conjugated goat anti-mouse Ab (Sigma, IL). Virus foci were visualized by the addition of TrueBlue HRP substrate (KPL, MD). Foci were counted and viral titers were calculated by standard methods.

## Supplementary References


1 Johnson, B. W., Russell, B. J. & Lanciotti, R. S. Serotype-specific detection of dengue viruses in a fourplex real-time reverse transcriptase PCR assay. *J Clin Microbiol* 43, 4977-4983, doi:10.1128/JCM.43.10.4977-4983.2005 (2005).

2 Santiago, G. A. *et al.* Analytical and clinical performance of the CDC real time RT-PCR assay for detection and typing of dengue virus. *PLoS Negl Trop Dis* 7, e2311, doi:10.1371/journal.pntd.0002311 (2013).

3 White, L. J. *et al.* An alphavirus vector-based tetravalent dengue vaccine induces a rapid and protective immune response in macaques that differs qualitatively from immunity induced by live virus infection. *J Virol* 87, 3409-3424, doi:10.1128/JVI.02298-12 (2013).
